# Cell Behavior of Primary Fibroblasts and Osteoblasts on Plasma-Treated Fluorinated Polymer Coated with Honeycomb Polystyrene

**DOI:** 10.3390/ma14040889

**Published:** 2021-02-13

**Authors:** Klára Fajstavrová, Silvie Rimpelová, Dominik Fajstavr, Václav Švorčík, Petr Slepička

**Affiliations:** 1 Department of Solid State Engineering, University of Chemistry and Technology Prague, Technická 5, 166 28 Prague, Czech Republic; klara.fajstavrova@vscht.cz (K.F.); dominik.fajstavr@vscht.cz (D.F.); vaclav.svorcik@vscht.cz (V.Š.); 2 Department of Biochemistry and Microbiology, University of Chemistry and Technology Prague, Technická 5, 166 28 Prague, Czech Republic

**Keywords:** polystyrene, cytocompatibility, cell viability, fluorinated ethylene propylene, plasma treatment, honeycomb-like pattern

## Abstract

The development of new biocompatible polymer substrates is still of interest to many research teams. We aimed to combine a plasma treatment of fluorinated ethylene propylene (FEP) substrate with a technique of improved phase separation. Plasma exposure served for substrate activation and modification of surface properties, such as roughness, chemistry, and wettability. The treated FEP substrate was applied for the growth of a honeycomb-like pattern from polystyrene solution. The properties of the pattern strongly depended on the primary plasma exposure of the FEP substrate. The physico-chemical properties such as changes of the surface chemistry, wettability, and morphology of the prepared pattern were determined. The cell response of primary fibroblasts and osteoblasts was studied on a honeycomb pattern. The prepared honeycomb-like pattern from polystyrene showed an increase in cell viability and a positive effect on cell adhesion and proliferation for both primary fibroblasts and osteoblasts.

## 1. Introduction

In 1991, an American research team led by Vacanti successfully implanted the first cells-seeded scaffold from a synthetic polymer to the human body [1]. Many research studies on this topic are published every year and have shown how artificial polymer substrates are promising candidates in tissue engineering [2]. The essential characteristics of the polymer scaffold are biocompatibility, high porosity with suitable pore array, high surface area, appropriate mechanical strength, and positive cell interaction (adhesion, proliferation, and differentiation) [3]. For the polymer substrates to be as attractive to cells as possible, it is desirable to modify their surface and, thereby, change their properties to be cell-attractive. Several techniques for surface optimization of materials, such as laser and plasma treatment, ion implantation, and carbon and metal nanoparticle grafting, and their positive effects on cell growth have been described [4,5,6]. Crucial surface characteristics regulating cell behavior have been discussed, mainly, surface chemistry, wettability, energy, morphology, roughness, electrical charge, and conductivity [7]. Individual cell types have specific requirements for the surface to which they adhere. For example, bone cells, osteoblasts, prefer a surface of relatively high roughness, though roughness higher than 2.19 µm inhibits osteoblastic adhesion, moderate surface hydrophilicity, and positive charge [8], as well as surface oxygen enrichment. Moreover, surface nanostructuring also improves bone cell adhesion [9]. Human osteoblasts vary in their size and shape, mostly having the size of 20–30 μm up to 50 μm [10]; they are sensitive to surface chemistry and topography on the nano-, micro-, and mesoscales [11,12].

The effect of surface roughness on the cell response depends strongly on the cell type and size [13]. The macro roughness (100 μm to 1 mm) can influence the behavior of larger cells (neurons) and usually does not restrict the adhesion and spreading of small cells [14]. At a micron and submicron scale (100 nm to 100 μm), many researchers have contradicting opinions on cell response to these surfaces. Some studies show obvious cell adhesion and proliferation [15,16] while others describe the negative effect of microroughness on cell behavior [17,18]. Small cells, such as endothelial cells, are sensitive to surface nanoroughness (less than 100 nm), which demonstrably improves their adhesion and growth [19]. The influence of nanotopography on the response and protein adsorption of a variety of cells has been discussed by Lord et. al. [20]. However, different biomaterials have different requirements for cell adhesion despite the same surface roughness. There is no general trend that describes the relationship between the surface roughness and the cell response; it always depends also on other physicochemical properties of the biomaterials and the specific cells and their phenotype [21]. The surface charge of the material strongly affects the cell behavior [22] and it can be regulated through the chemical functional groups in the polymer chains. The positively charged surface has been proved to provide significantly better cell adhesion to the material surface or the cell-cell interaction than the negatively charged surface [23,24,25]. The reason is the negative charge of extracellular matrix (ECM) proteins mediating cell adhesion. It has been shown that the shape and arrangement of ECM proteins have a greater effect on the resulting cell adhesion than their amount [26]. A similar amount of protein osteopontin was adsorbed on the substrate with a positive charge (containing –NH_2_ groups) and negative charge (containing –COOH groups), but the resulting number and the distribution of seeded aortic endothelial cells were higher on the positively charged substrate [27].

Based on the aforementioned requirements on a polymer scaffold for osteoblasts, a positive response of these cells can be initiated by, for example, a porous structure called a honeycomb-like pattern (HCP) [28]. This pattern can be prepared from different types of polymers [29] and can be either a layer [30] or a self-supporting film [31]. With a suitable pore size, arrangement, and shape it can serve as a suitable environment for cell growth [32]. The appearance of the pores is influenced by preparation conditions such as the material type, solvent, concentration of polymer solution, additives, and humidity [33]. Wu et al. [34] compared the behavior of mouse preosteoblasts (MC3T3-E1) on a poly(ε-caprolactone) substrate with HCP and a flat control without a surface structure. The cells copied the HCP and showed augmented adhesion and proliferation compared to the control without a surface structure. The advantage of the honeycomb-like structure in the application as a polymer scaffold is its spatial control or directional guidance. The effect of honeycomb-like microtopography on osteospecific and myospecific differentiation of human mesenchymal stem cells was examined by Kawano et al. [35].

The first material used for the fabrication of layers with HCP using a breath figure technique was polystyrene (PS) [36]. This versatile polymer has been a basic cell platform for more than 50 years. The reason why this synthetic aromatic polymer is used as a cell matrix is its cost-effectiveness, unique formability, non-toxicity, and low crystallinity. PS films can be prepared using simple techniques such as dip coating and spin coating [37], facile functionalization with different functional groups [38], or patterning of the surface for cytocompatibility enhancement [39,40]. The development of PS as a material for cell culture was described by Lerman et al. [41]. The question of how to facilitate cell behavior on commercial tissue culture PS has been addressed by Huang et al. [42]. His team invented 3D honeycomb-patterned Petri dishes with incorporated polyethylene glycol (PEG) using a direct breath figure in which HCP is a part of the substrate and no additional polymer solution is needed. The effect of surface morphology on cell orientation has also been studied, as well. Biomaterials with different percentages of PEG were also used as antimicrobial materials [43].

A research study on argon plasma treatment of polymer substrates and its positive effect on adhesion and proliferation of various cell types was presented in [44]. This simple approach can change the topography and other surface physico-chemical properties which play a crucial role in cell attachment. Our group has focused on plasma treatment of fluorinated ethylene propylene (FEP), which is a polymer with high-temperature stability and excellent chemical resistance [45]. The effect of individual plasma treatment parameters on the surface and biological properties of this polymer is described in refs. [46,47,48]. Another idea was to combine a durable biocompatible substrate (FEP) with a biocompatible polymer porous layer to create an attractive 2D/3D cell environment. To morphologically control the polymer film, we chose an improved phase separation method. Using this technique, we have successfully prepared an HCP layer from poly-L-lactic acid (PLLA) and acetate cellulose on plasma-modified FEP [49]. As previously mentioned, PS is a widely used material for tissue culture and it was the first candidate for the construction of HCP structures. Therefore, our aim was to create a porous film from PS and subsequently to test the designed substrate in vitro for potential biomedical applications.

## 2. Materials and Methods

### 2.1. Materials and Chemicals

A commercial polystyrene foil (PS; 50 μm thickness, biaxially oriented, the density of 1.05 g cm^−3^, received from Goodfellow Cambridge Ltd., Huntington, UK). Chloroform (CHCl_3_; stabilized with 1% ethanol A.G., M_r_ 119.38, supplied by Penta, Prague, Czech Republic) and methanol (MeOH; for HPLC, M_r_ 32.04, supplied by Penta, Prague, Czech Republic) were used as solvents. FEP foil (50 μm thickness, density of 2.15 g cm^−3^, received from Goodfellow Cambridge Ltd., Huntington, UK) was used as a substrate.

### 2.2. Preparation of Substrates

The surface of the FEP foils was activated using argon plasma discharge [50] with a Balzers SCD 050 device (Baltec, Balzers, Liechtenstein). The conditions during activation were the following: gas pressure 10 Pa, room temperature, input power of 3 and 8 W, and exposure time of 40 and 240 s. Prepared substrates were dipped into the homogenous ternary mixture of chloroform, methanol (100 mL, volume ratio 90/10), and PS (2 g) for 10 s, and then, it was kept in the air and RT to complete evaporation of the solvents.

### 2.3. Characterization of Substrates

The surface topographies of modified substrates were examined using atomic force microscopy (AFM) with Dimension ICON (Bruker Corp., Billerica, MA, USA). The surface was measured in Scan-Assyst mode using nitride lever SCANASYST-AIR (Bruker Corp., Billerica, MA, USA) with Si tip (spring constant of 0.4 N m^−1^). NanoScope Analysis software was used for processing of data. The particular characteristics such as morphology, size, and shape of pores of the prepared HCP structures were characterized using scanning electron microscopy (SEM) with a LYRA3 (Tescan, Brno, Czech Republic). The applied acceleration voltage for SEM was 10 kV. The metallization of substrates was realized via platinum sputtering, with a deposited Pt thickness of 20 nm (Quorum Q300T, Laughton, UK).

The thickness of the ablated layer after plasma exposure and the thickness of the coated PS layer was measured via gravimetric analysis using microbalance Mettler Toledo UMX2 (Mettler-Toledo, Columbus, OH, USA). To avoid fluctuations in mass values, the charge on the sample surface was reduced before weighing with the radio-frequency field depolarization gate. The thickness of the layers was calculated from the values of the weight gains/increments.

The surface chemistry of the prepared samples was examined with X-ray photoelectron spectroscopy (XPS) using spectrometer ESCAProbeP (Scienta Omicron GmbH, Taunusstein, Germany). The source was a monochromatic X-ray at the energy of 1486.7 eV. CasaXPS software was used. As a secondary method for the analysis of elemental concentration, energy-dispersive X-ray spectroscopy (EDS) using an F-MaxN analyzer (Oxford Instruments, Abingdon, UK) and SDD detector (Oxford Instruments, Abingdon, UK) was used. The applied acceleration voltage for EDS was 10 kV. The metallization of substrates was realized via platinum sputtering, with a deposited Pt thickness of 20 nm (Quorum Q300T, Laughton, UK).

The wettability changes of treated and prepared surfaces were studied by use of contact angle determination. The Surface Energy Evaluation System (SEE System, Advex Instruments, Brno, Czech Republic) was applied for this study; 8 drops of 8.0 ± 0.2 µL volume of distilled water were applied to the sample using an automatic pipette with subsequent photograph analysis.

### 2.4. Cell Culture

The cell lines used in this study were lung fibroblasts MRC-5 (human primary cells) and U-2 OS cells (human cells from osteosarcoma), which were supplied by the ATCC (American Tissue Culture Collection, Manassas, VA, USA). MRC-5 cells were cultivated in MEM (minimal essential media; Thermo-Fisher Scientific, Waltham, MA, USA) and U-2 OS cells in high glucose DMEM (Dulbecco’s modified Eagle’s medium; Thermo-Fisher Scientific, Waltham, MA, USA) both with 2 mM L-Glutamine (Sigma, St. Louis, MO, USA) and 10% FBS (fetal bovine serum; Thermo-Fisher Scientific, Waltham, MA, USA). The cells were passaged two to three times a week so that they remained in exponential growth. The cultivation conditions were 5% CO_2_, 37 °C, and 95% humidity. Cell medium was changed for 5 mL of PBS (phosphate-buffered saline; pH 7.4, prepared in the laboratory); then, the PBS was removed and the cells were detached using trypsin-EDTA solution (1 mL) (Thermo-Fisher Scientific, Waltham, MA, USA). After 3 min, 5 mL of media was added, and an aliquot of 500 µL of cell suspension was transferred in 10 mL of fresh media on a new Petri dish. As for the MRC-5 cells, the experiments were performed until the 16th number of cell divisions until which the primary phenotype should be maintained. Experiments with U-2 OS cells were done between the 6th and 10th passage.

### 2.5. Cell Viability

Viability of U-2OS and MRC-5 cells adhered and grown on the tested samples was determined using a WST-1 test, as described in detail in ref. [51]. The WST-1 method lies in transformation of a WST-1 agent to formazan product, which can be monitored spectrophotometrically at 450 nm. The examined materials were sterilized with 70% ethanol for 40 min and inserted into 12-well dishes (Ø 2.14 cm, VWR, Radnor, PA, USA,), washed with PBS, and weighted using poly(methyl)methacrylate cylinders (Zenit, Prague, Czech Republic). Then, 15,000 cells per one cm^2^ were transferred onto the tested substrates in a total in 1 mL of media (MEM for MRC-5 and DMEM for U-2 OS). Samples were done in triplicates. After cultivation (24, 48, and 72 h time points), the medium was changed for PBS and then replaced with solution containing WST-1 (1:20) in DMEM without phenol red. After 2 h incubation, 100 μL aliquots of the culture media with formed formazan were transferred to wells of 96-well plates in quadruplicates and subjected to spectrophotometric measurement at 450 nm (reference 650 nm). Cells grown on tissue culture polystyrene (PS, 12 wells) and untreated FEP were utilized as controls.

### 2.6. Cell Seeding for Microscopy Analysis

U-2 OS and MRC-5 cell morphology and proliferation were followed by fluorescence microscopy. For this purpose, the cells were inoculated as described in Section 2.5. Then, at each time point (1, 3, and 6 days of cultivation), the cells were twice washed with PBS and subjected to fixation as described in ref. [52]; i.e., the fixation solution was composed of 4% of formaldehyde for tissue culture (Thermo-Fisher Scientific, Waltham, MA, USA) in PBS. After 15 min of fixation in the dark, the fixative was changed for PBS and then again washed with PBS. After that, the cell F-actin was labelled with phalloidin-Attto 488 (Sigma, St. Louis, MO, USA; 2 μg mL^−1^) and cell nuclei with DAPI (4′,6-diamidino-2-phenylindole dilactate; Sigma, St. Louis, MO, USA; 0.5 μg mL^−1^). The staining solution was changed for PBS, washed using PBS again, and the samples were analyzed using microscopy.

### 2.7. Fluorescence Microscopy of MRC-5 and U-2 OS Cells

Fluorescence microscopy analysis of U-2 OS and MRC-5 cells cultivated on the examined substrates was achieved using an inverse fluorescence microscope (Olympus IX-81, Tokio, Japan) with xCellence software. Cells of both cell lines were monitored at magnifications of 100× (10× objective, NA = 0.30), 200× (20× objective, NA = 0.45, NA–numerical aperture), and 400× (40× objective, NA = 0.60) using an EM-CCD camera (Hamamatsu, Honshu, Japan). F-actin and nuclei of the cells were monitored using a triple filter DAPI/FITC/TRITC (Olympus, Tokio, Japan). The fluorescence cell images were corrected for background, and DAPI and FITC channels were merged.

### 2.8. SEM of MRC-5 and U-2 OS Cells

To detect detailed MRC-5 and U-2 OS cell morphology on the evaluated substrates, scanning electron microscopy (SEM) was used. Cells of both cell lines were seeded in the same way as in Section 2.5 but only duplicates were prepared. After 72 h of cultivation, rinsing (twice) with PBS was done followed by fixation with Karnovsky fixative (prepared in the laboratory) in a cacodylate buffer (prepared in the laboratory). Then, the samples were dehydrated with 50, 70, 80, and 90% ethanol in deionized water, after which double rinsing with 99.9% ethanol followed. Each step took 15 min, similarly as in ref. [46]. The dehydration was completed with immersion of the samples into hexamethyldisilazane (Sigma, St. Louis, MO, USA) for 15 min, twice. Additional sample drying was achieved overnight at 30 °C. The acceleration voltage applied during the SEM analysis was 10 kV. The samples were subjected to coating with a Pt conductive layer with a 20 nm thickness using a diode sputtering method (Quorum Q300T, Laughton, UK). Cells on glass coverslips were used as controls.

## 3. Results

**Figure 1:**
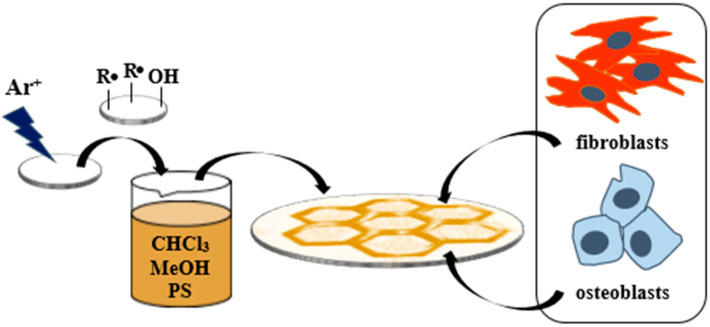
Scheme of the preparation of a polymer scaffold for cell culture.

This work aimed to fabricate a biocompatible polymer matrix for cell adhesion. As a substrate for the preparation of the polymer scaffold, a polymer foil from FEP was used. The foil was modified with argon plasma at different powers (3 and 8 W) and at different exposure times (40 and 240 s). The plasma treatment was performed to increase material biocompatibility [48] and to attach a PS layer [49]. Based on improved phase separation, PS HCPs were successfully formed on all modified FEP substrates. Methanol plays an important role here—it simulates humid conditions (the classic breath figure method occurs at elevated humidity), induces phase separation, and stabilizes the droplets that form a porous structure [53]. Subsequently, two types of cells were cultured on these matrices-human primary fibroblasts (MRC-5) and human osteoblasts (U-2 OS). The scheme of the sample preparation process is depicted in Figure 1.

### 3.1. Surface Morphology, Roughness, and Surface Area

**Figure 2:**
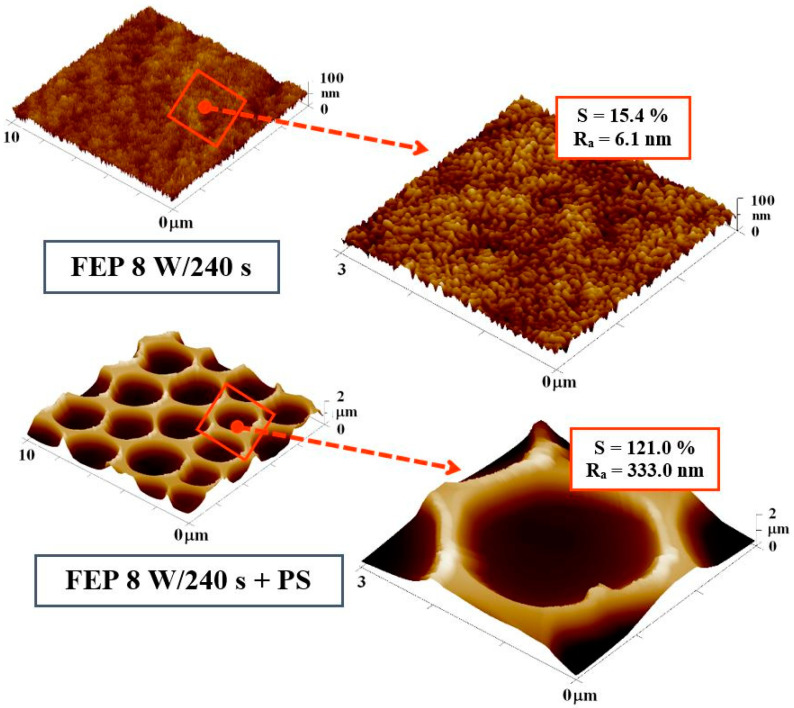
Atomic force microscopy (AFM) scans (10 × 10 µm^2^) of plasma-modified fluorinated ethylene propylene (FEP) (8 W/240 s) and subsequently coated with polystyrene (PS) creating a honeycomb-like pattern (HCP) (8 W/240 s + PS) and corresponding detailed images (3 × 3 µm^2^). R_a_ represents the average of the deviations from the center plane of the sample and S represents a specific surface area.

Surface morphology and roughness of pristine FEP and the effect of plasma treatment on an increase in surface topography and roughness is demonstrated in ref. [46]. Figure 2 shows a comparison of the FEP surface morphology before and after PS deposition. In our previous study, promising conditions suitable for cell adhesion were determined for various plasma treatment set-ups, the most effective of which were at longer exposure times such as 240 s (for the plasma-treated FEP and also for subsequently coated FEP) [49]. AFM images of FEP samples treated at high plasma power (8 W) and long exposure time (240 s) were selected for AFM analysis. The enlarged image of the plasma-modified sample in Figure 2 shows a wrinkled structure on the FEP surface caused probably by enhanced ablation of the amorphous phase of FEP, as discussed in ref. [49]. After the formation of the PS film with HCP, a significant increase in the surface area (121.0%) and roughness (R_a_; 333.0 nm) was detected. Both factors significantly contribute to the attractiveness of the polymer substrate for cell attachment. The pores are circular, regularly arranged, and the size of one pore is about 3 µm. The same trend can be followed when comparing with samples treated with plasma at 3 W.

### 3.2. The Thickness of Prepared Substrates

**Figure 3:**
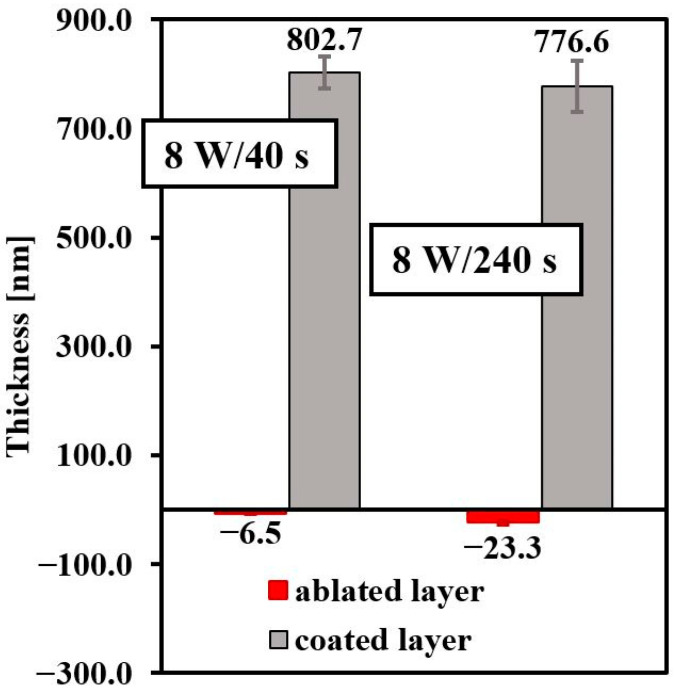
The thickness of an ablated fluorinated ethylene propylene layer after plasma treatment (8 W) and coated with a polystyrene (PS) layer in dependence on exposure time (40 and 240 s).

Plasma treatment causes ablation of the polymer surface and thereby changes the material surface properties [50]. The thickness of the FEP ablated layer was measured using gravimetry. A comparison of the thickness of the ablated layer on FEP at 3 and 8 W was reported by our group in ref. [46]. We showed that the higher power of the plasma discharge (8 W) caused more pronounced polymer ablation. In Figure 3, there is a red diagram representing the thickness of the ablated layer depending on the plasma exposure time (40 s and 240 s) for plasma-treated samples at higher power (8 W). As we expected, with a longer modification time (240 s), there was a bigger material loss (−23.3 nm). After the application of the PS layer of FEP, solvent evaporation, HCP formation, and the thickness of the prepared films were also determined using gravimetric analysis. The thickness was dependent on the length of the plasma exposure (represented by gray diagrams). The results show that we achieved preparation of a thick polystyrene layer (802.7 nm) on FEP exposed to plasma treatment for 40 s.

### 3.3. Surface Chemistry

**Figure 4:**
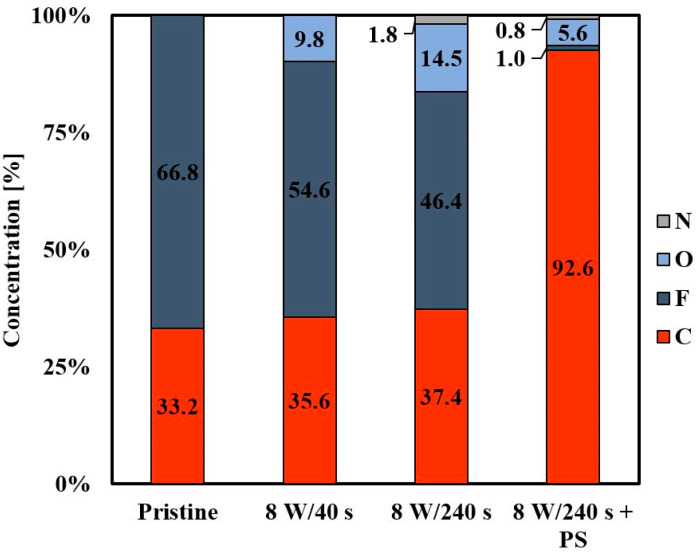
Concentration of elements (C, F, O, N) on pristine fluorinated ethylene propylene (FEP), plasma-treated FEP (8 W/40 s and 8 W/240 s), and subsequently coated with a polystyrene (PS) layer (8 W/240 s + PS).

The elemental composition on the sample surface measured using XPS (under the take-off angle of 14°) can be seen in Figure 4. The unmodified FEP contains carbon (33.2%) and fluorine (66.8%) in its chain. Oxygen occurs in samples after plasma modification. The presence of oxygen is caused by the cleavage of polymer chains, the formation of radicals, and new oxygen functional groups. We observed that a longer plasma modification time (240 s) induced higher oxygen representation and less fluorine content on the FEP-treated surface. The occurrence of a small amount of nitrogen on the polymer surface after the plasma treatment for 240 s was detected. Atmospheric nitrogen reacts with radicals on activated FEP and creates a nitrogen functional group. If we look at the elemental composition of the FEP surface after applying the PS layer, the carbon content rapidly increased (92.6%) and the fluorine and oxygen content were reduced to a minimum. This result confirms the successful formation of a PS film since PS contains mainly carbon in its chain. In general, higher oxygen content increases the attractiveness of a substrate to cell adhesion. By comparing the surface chemistry before the application of PS and after the formation of PS HCP, we conclude that a more attractive substrate will be plasma-treated FEP containing higher amounts of oxygen. However, other factors, such as surface morphology and roughness, play a significant role here.

**Figure 5:**
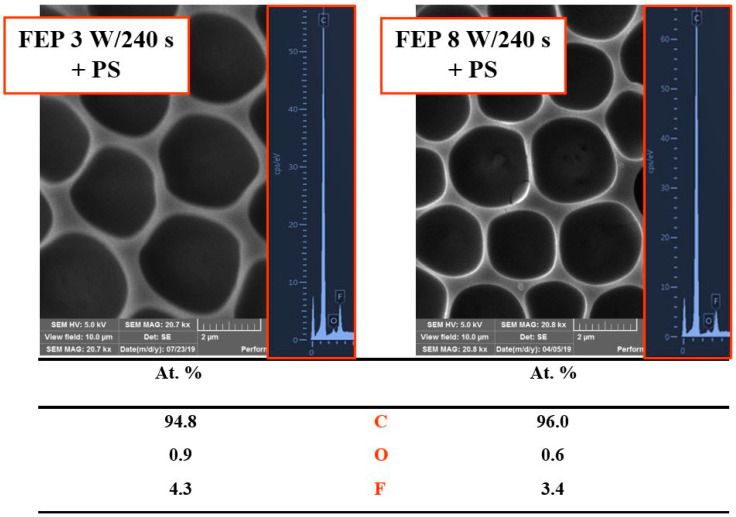
Scanning electron microscopy images (10 × 10 µm^2^) of plasma-modified fluorinated ethylene propylene (FEP) treated at different plasma discharges (3 and 8 W) with a polystyrene (PS) layer forming a honeycomb-like pattern. On the right side, corresponding energy-dispersive X-ray spectroscopy graphs and a table of element concentration on the surface are depicted.

The effect of different plasma powers (3 and 8 W) at the same plasma modification time (240 s) on the pore size and shape and the corresponding chemical surface composition is shown in Figure 5. The pores had a circular shape and size of 2–3 µm in diameter. The results of the EDS analysis are shown both graphically and numerically. This analysis can acquire elemental composition from a greater depth (approx. up to 100 nm from the surface). Therefore, compared to XPS results, a higher amount of fluorine contained in the FEP chains was detected. The depth of acquisition is also connected with the surface morphology of analyzed samples. The oxygen in the EDS analysis is based mostly on a determination of the surface oxygen of plasma-treated perfluorinated surface. Even though only a low amount was detected with EDS/EDX, it confirms the results from XPS and also wettability determination, that perfluorinated surface is activated by argon plasma (the activation partially remains even after honeycomb pattern formation) and the surface physico-chemical changes play an important role in the formation of the pattern. The values listed in the table show that the chemical composition did not change significantly when using different powers of plasma discharge.

### 3.4. Surface Wettability and Aging

**Figure 6:**
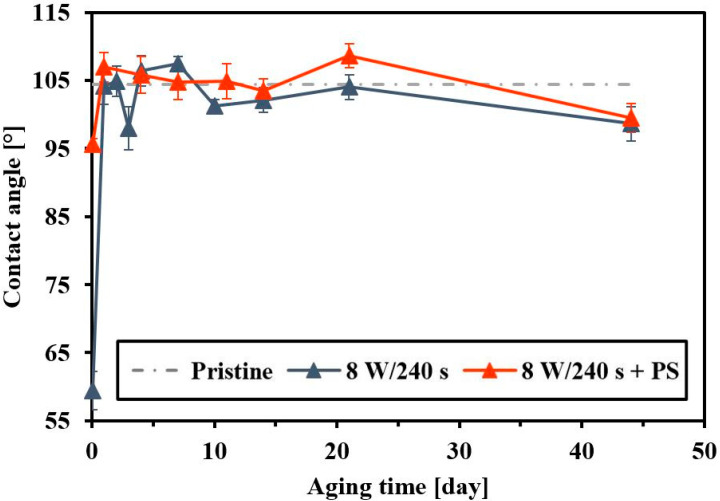
Contact angles and aging of pristine fluorinated ethylene propylene (FEP), plasma-treated FEP (8 W/240 s), and subsequently coated with a polystyrene (PS) layer (8 W/240 s + PS).

Surface chemistry is closely related to another important factor that characterizes the surface of a material, surface wettability. The wettability was determined by goniometry, by measuring the contact angle on a sessile water drop on selected samples. Depending on the conditions of the FEP plasma treatment (8 W, 240 s) and PS coating of the substrate, we measured changes in contact angles (see Figure 6). The contact angles were measured immediately after FEP plasma modification after so-called polymer surface aging (after 14 days). The dashed line shows the value of the unmodified FEP substrate (104.4°). Immediately after modification of FEP in plasma, the contact angle sharply dropped (59.8°) and the wettable surface developed. This was probably caused by cleavage of the polymer chain, the formation of radicals that react with air oxygen and create hydrophilic functional groups. A connection of wettability changes with surface chemistry, for which higher oxygen content causes a decrease in a contact angle and increased substrate hydrophilicity, was determined, the values in the first stages of aging being slightly different compared to [46]. During 14 days from modification, there was a sharp increase in the water contact angle (104.1°); after this period only mild fluctuations in the contact angle were observed. The hydrophilic oxygen groups are turned inward and the oxygen diffused into the internal volume of the polymer, making the surface more hydrophobic. Comparison of the contact angles of pristine FEP and plasma-treated FEP revealed that the change occurred immediately after the modification, otherwise, the following values were similar. Despite the surface polarity, cell adhesion is largely affected also by other parameters such as surface charge and morphology. Immediately after the formation of the PS layer on the FEP, the contact angle was lower than on the pristine FEP but higher than on the plasma-treated substrate. As demonstrated by the XPS analysis, oxygen dropped after the formation of HCP, making the surface more hydrophobic. After the aging of the sample, the value of the contact angle of the honeycomb pattern on plasma-treated FEP (8 W/240 s + PS) within the measurement error was similar as for pristine and plasma-treated FEP.

**Figure 7:**
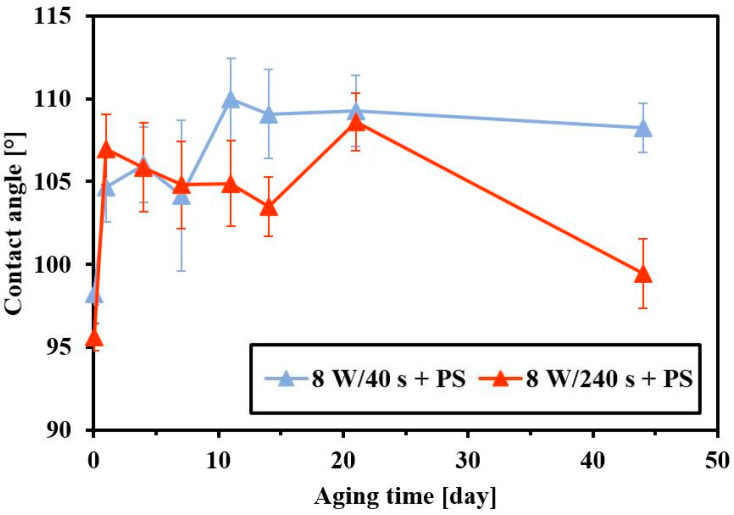
Contact angles and aging of fluorinated ethylene propylene (FEP) samples after plasma modification (8 W) and subsequently coated with a polystyrene (PS) layer in dependence on plasma exposure time (40 s and 240 s).

Figure 7 shows changes in contact angles in dependence on the length of the FEP plasma modification. It is apparent that longer exposure times of plasma treatment created a more hydrophilic surface. The effect of different plasma modification powers (3 and 8 W) on the wettability and aging of the FEP surface is discussed in ref. [31]. From previous measurements, it can be stated that PS HCP formation at different plasma powers does not affect the contact angle significantly.

### 3.5. Cytocompatibility

**Figure 8:**
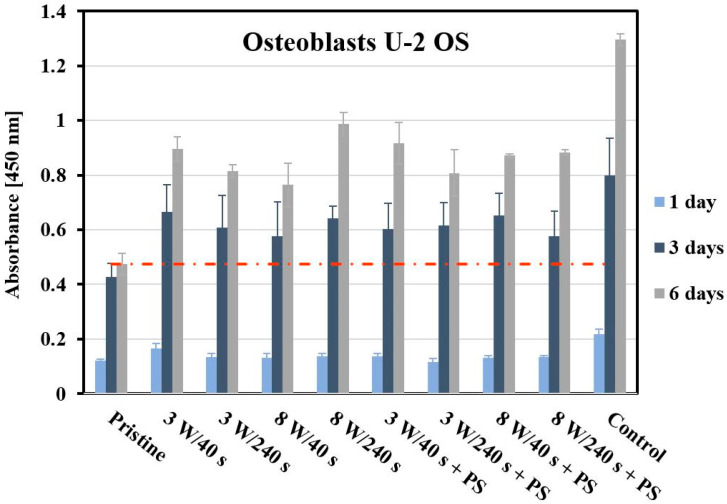
Metabolic activity of human osteoblasts (U-2 OS) growing on the studied FEP substrates. Cell metabolic activity was determined spectrophotometrically using WST-1 assay at 450 nm at 1, 3, and 6 days post-seeding. As a control, glass coverslips were used. The error bars represent the standard error of the mean of three replicates.

The interaction between polymer matrices and cell cultures determines the cytocompatibility of the material. Ar plasma treatment of an otherwise inert FEP polymer positively affects the adhesion and viability of human keratinocytes (HaCaT), as previously reported [46,47]. In this study, we evaluated the growth, viability, and morphology of two cell types, human osteoblasts (U-2 OS) and primary fibroblasts (MRC-5) cultivated on FEP and PS micropattern. The U-2 OS cell response was monitored 1, 3, and 6 days post-seeding using the WST-1 method (see Figure 8). This test is based on the determination of the cells’ metabolic activity. A glass coverslip (control) served as a control sample. The first day after seeding, the metabolic activity of U-2 OS cells was slightly lower on all samples in comparison to the control substrate. However, there were no significant differences in cell viability between treated and untreated substrates. After 3 days, pronounced cell proliferation was evident, cell metabolic activity was significantly increased to values close to the control sample. The lowest cell viability was detected on the pristine FEP, however, the difference compared to HCP samples after 3 days was not as significant as after 6 days.

At six days post-seeding, the absorbance values of WST-1 were quite similar for plasma-treated substrates and substrates coated with PS. At six days post-seeding, there was an augmentation in metabolic activity of osteoblasts growing on all tested substrates. On the other hand, the viability of the U-2 OS cells growing on the control samples was significantly pronounced in comparison to pristine FEP, but also to other studied samples. Regarding only the plasma-treated samples, the best results in terms of cell viability were shown for samples modified at 8 W for 240 s. At higher power (8 W), the results slightly differed. With a shorter exposure time (40 s), there was a moderate increase in the metabolic activity of U-2 OS cells growing on this substrate. On the other hand, there was a decrease in cells growing on the 8 W/240 s sample. FEP surface treatment (plasma modification and deposition of the PS layer) led to a significant improvement of cytocompatibility (especially after 6 days). 

**Figure 9:**
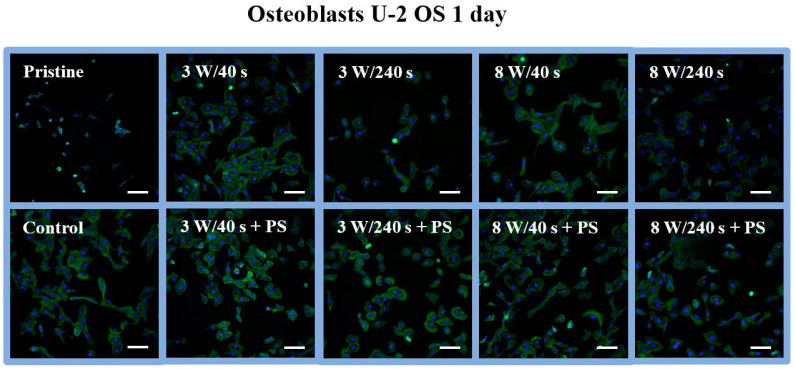
Fluorescence microscopy images of human osteoblasts (U-2 OS) at 1 day post-seeding on FEP (pristine), FEP matrices treated using plasma with 3 and 8 W (40 and 240 s) and subsequently coated with a honeycomb-like pattern formed from PS. As a control, glass coverslips were used. Nuclei stained with DAPI are in blue, F-actin labeled with phalloidin-Atto 488 is in green. The scale bars represent 100 µm.

**Figure 10:**
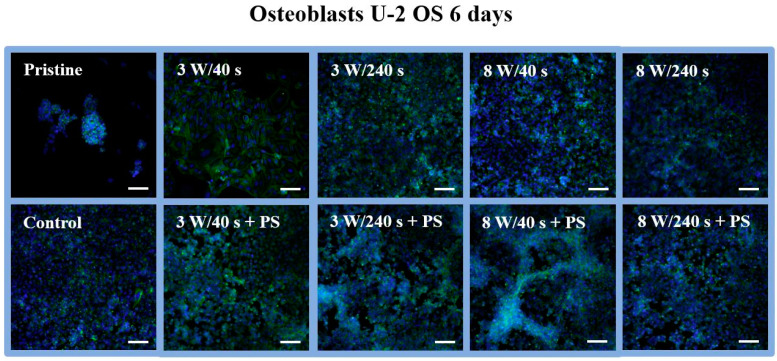
Fluorescence microscopy images of human osteoblasts (U-2 OS) at 6 days post-seeding on FEP (pristine), FEP matrices treated using plasma with 3 and 8 W (40 and 240 s) and subsequently coated with a honeycomb-like pattern formed from PS. As a control, a glass coverslip was used. Nuclei stained with DAPI are in blue, F-actin labeled with phalloidin-Atto 488 is in green. The scale bars represent 100 µm.

In order to further evaluate cell response, morphology, and spreading on the tested substrates, samples with U-2 OS cells cultivated for 1 (Figure 9) and 6 days (Figure 10) were selected. Cell behavior was captured using a fluorescence microscope. After day 1, a mixture of round and only partially spread cells, not characteristic for osteoblasts, was observed at the pristine FEP (Figure 9). Contrary to that, the morphology of cells growing on control samples corresponded to the physiological shape of osteoblasts. At all plasma-modified samples, the cells had the same shape as at of the control, but they were inhomogeneously distributed. We even observed dividing cells on selected substrates (3 W/240 s, 8 W/40 s). Probably, part of the cell population copied the porous structure of the HCP. As we could observe on plasma-treated samples, the cells were unevenly distributed. The surface coverage by cells was similar to that of the control on substrate modified for 8 W/240 s with an applied HCP layer (Figure 9). In addition, on the 8 W/240 s sample, the cells exhibited the optimal shape, and dividing cells were also observed. Figure 10 shows fluorescence microscopy images of proliferating osteoblasts 6 days after cultivation. On unmodified FEP, large clusters of growing round cells on a maximum of one-fifth of the total sample area were detected. This may be due to the high hydrophobicity of the polymer surface, which prevents cell adhesion. As expected, the control substrate was completely covered with osteoblasts. The shape of these cells was no longer distinguishable. The plasma-modified samples were also fully covered with U-2 OS cells as on the control, except for the 3 W/40 s sample. On this sample, the cells grew nicely elongated and spread out but did not cover the entire substrate. Regarding samples with HCP, the number of cells was lower compared to plasma-modified samples (Figure 8). Space without cells (around 10% of the sample area) is apparent on the images from fluorescence microscopy (Figure 9).

**Figure 11:**
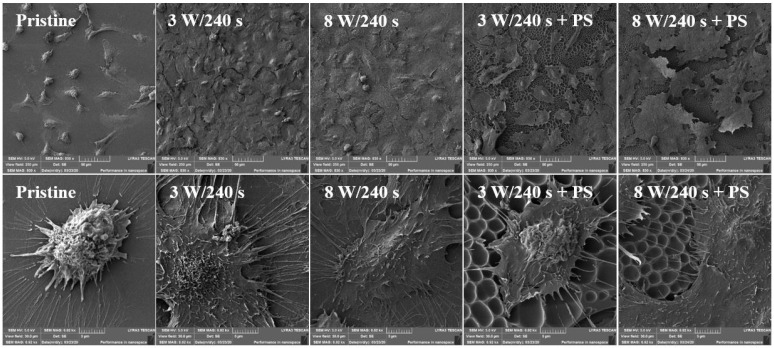
Scanning electron microscopy images of human osteoblasts (U-2 OS) at 6 days post-seeding on FEP (pristine), FEP matrices treated using plasma with 3 W and 8 W (240 s) and subsequently coated with a honeycomb-like pattern formed from PS. The upper line represents a 250 × 250 µm^2^ scan, the bottom line a detailed scan with an area of 30 × 30 µm^2^.

The sample surface morphology was studied using scanning electron microscopy. Results only for selected samples are shown to confirm the constructed HCPs on plasma-activated FEP samples. The SEM analysis provided detailed information on the U-2 OS morphology and cell-cell and material-cell contacts (Figure 11). For SEM, samples modified at 3 and 8 W for 240 s and coated with a PS film were selected. The pristine FEP was chosen for comparison. The morphology of human osteoblasts on the pristine FEP substrate differed significantly from cells growing on other examined samples, which confirmed the results from fluorescence microscopy. The most pronounced spreading of U-2 OS cells on the examined samples was detected on plasma-modified FEP (3 W, 8 W). The cells were flat, interacted with each other, and covered the entire material surface. When comparing the samples with an applied PS layer, the cells prospered more on the substrate modified at higher power (8 W). The data are in agreement with those from fluorescence microscopy (Figure 9) as well as from our previous in vitro tests of the HCP layer formed from PLLA [54].

**Figure 12:**
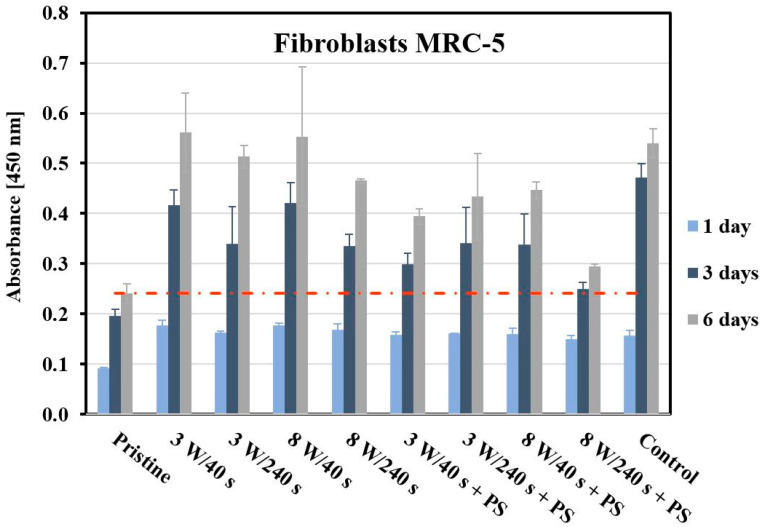
Metabolic activity of primary human fibroblasts (MRC-5) growing on the studied FEP substrates. Cell metabolic activity was determined spectrophotometrically using WST-1 assay at 450 nm at 1, 3, and 6 days post-seeding. As a control, glass coverslips were used. The error bars represent the standard error of the mean of three replicates.

Different cell types, such as bone cells or fibroblasts, each require treatment leading to different surface properties, e.g., is it expected for bone cells to proliferate on surfaces with higher effective roughness. The metabolic activity of fibroblasts on surfaces with increased roughness and a specific hexagonal pattern was monitored in this work. The MRC-5 cell metabolic activity was monitored after 1, 3, and 6 days post-seeding using the WST-1 method. Figure 12 shows the viability of primary human fibroblasts growing on pristine FEP, FEP treated using 3 W and 8 W plasma (40 and 240 s), and FEP with a polystyrene HCP structure, in contrast to a standard glass coverslip used as a control. The cellular behavior of another type of primary fibroblasts (human dermal fibroblast, HDF) on Ar plasma-treated FEP was studied in ref. [48]. The results showed improved proliferation and spreading of HDFs on plasma-modified FEP. If we compare the values of metabolic activity on untreated FEP and plasma-treated FEP after the first day from cultivation with the data in ref. [48], the results are very similar. A slightly different cellular behavior was evident on the pristine FEP, on which the HDF metabolic activity was comparable to on plasma-modified samples. In contrast, MRC-5 cells grew on unmodified FEP almost half less than on the other samples. After 3 and 6 days, there was an increase in the number of proliferated cells on all monitored substrates. Nevertheless, MRC-5 cells adapted better to the treated surface than to the original FEP. On the sample treated at 8 W, the metabolic activity of both osteoblasts and HDF cells [48] increased with longer exposure time. The opposite trend occurred with MRC-5 cells, on which more cells proliferated on the 8 W/40 s sample than on the 8 W/240 s sample. When evaluating MRC-5 cells, we can observe a clear difference between the metabolic activity of cells on plasma-modified samples and samples with an established HCP structure. Plasma modification was very attractive for the MRC-5 cells, which grew on these surfaces to a comparable extent as on the control (after 6 days). Decreased metabolic activity of MRC-5 cells was detected on samples with a HCP structure. Cells grown on the 8 W/240 s samples with PS, the metabolic activity of which approached pristine FEP values, proliferated the least.

**Figure 13:**
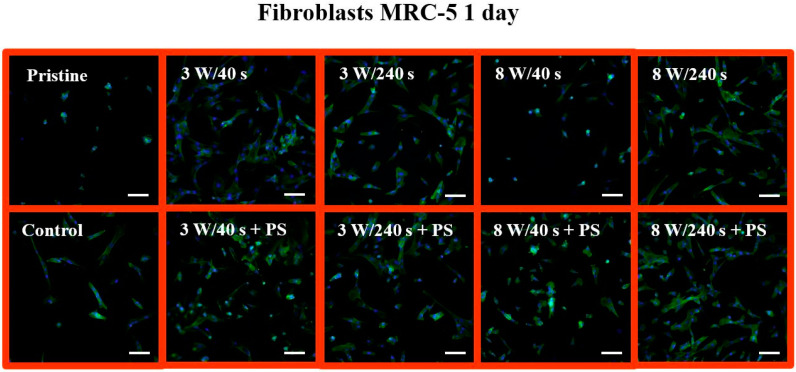
Fluorescence microscopy images of primary human fibroblasts (MRC-5) at 1 day post-seeding on FEP (Pristine), FEP matrices treated using plasma with 3 and 8 W (40 and 240 s) and subsequently coated with a honeycomb-like pattern formed from PS. As a control, a glass coverslip was used. Nuclei stained with DAPI are in blue, F-actin labeled with phalloidin-Atto 488 is in green. The scale bars represent 100 µm.

**Figure 14:**
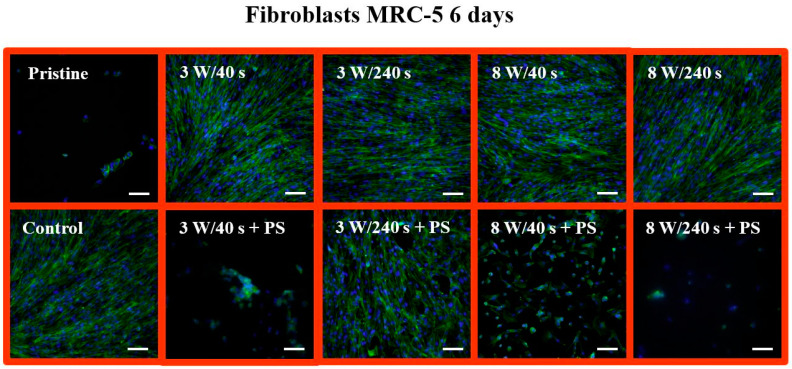
Fluorescence microscopy images of primary human fibroblasts (MRC-5) at 6 days post-seeding on FEP (pristine), FEP matrices treated using plasma with 3 and 8 W (40 and 240 s) and subsequently coated with a honeycomb-like pattern formed from PS. As a control, a glass coverslip was used. Nuclei stained with DAPI are in blue, F-actin labeled with phalloidin-Atto 488 is in green. The scale bars represent 100 µm.

For optimal detection of surface morphology, analysis of fluorescence microscopy images was performed. The shape and proliferation of MRC-5 cells on all samples were studied using fluorescence microscopy (Figure 13). The MRC-5 cells at 24 h post-seeding followed a similar trend as on untreated FEP, similarly as observed for U-2 OS cells (Figure 11). The cells had a round shape and not many of them adhered. On controls, elongated fibroblasts were observed. According to the images, the fewest cells grew on a sample treated with 8 W/40 s. A portion of the cells had an elongated shape, and the other portion had a round shape and connected in clusters as on the pristine FEP. In contrast, we see that the cells exhibited similar metabolic activity on this substrate as on the other samples. Another situation occurred after the application of a PS film. The cells had an irregular shape (some were triangular, semicircular, others were squared), but we can see a higher amount of proliferated MRC-5 cells compared to the control. The Ar plasma pre-treatment lasting for 240 s led to attachment of standard elongated fibroblasts and irregular cells. The progress in the proliferation and spread of MRC-5 cells on the studied materials is shown in Figure 14. We selected images of samples at 6 days post-seeding. Comparable results can be seen on all plasma-activated FEP and control glass, where the entire surface was covered with elongated fibroblasts. In contrast, small clusters of round cells were formed on untreated FEP, similar to osteoblasts. The surface with HCP also created unfavorable conditions for MRC-5 cells as pristine FEP, except for the 3 W/240 s sample. This substrate was almost entirely covered by MRC-5 cells, but not with a completely typical shape for fibroblasts. In addition, the cells grew chaotically in all directions and presumably adapted to the honeycomb HCP-like structure.

**Figure 15:**
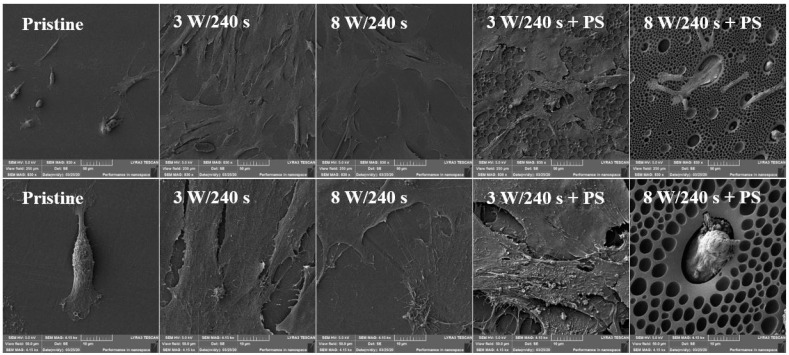
Scanning electron microscopy images of primary human fibroblasts (MRC-5) at 6 days post-seeding on FEP (pristine), FEP matrices treated using plasma with 3 W and 8 W (240 s) and subsequently coated with a honeycomb-like pattern formed from PS. The upper line represents a 250 × 250 µm^2^ scan, the bottom line a detailed scan with an area of 30 × 30 µm^2^.

For SEM analysis, samples modified at 3 and 8 W for 240 s and subsequently coated with a PS film were selected. From Figure 15, it is apparent that the surface morphology of MRC-5 cells on pristine FEP and 8 W/240 s + PS sample was different in comparison to other studied substrates. The cells were poorly spread with little cell filopodia attached to the surface. On the contrary, samples treated only using plasma discharge (both 3 and 8 W with the exposure of 240 s) and samples treated with lower plasma power and subsequently covered with a polystyrene pattern exhibited better results in terms of both cell spreading and cell number on the material surface.

## 4. Discussion

Crucial surface characteristics regulating cell behavior are surface chemistry, wettability, energy, morphology, roughness, electrical charge, and conductivity [7]. Our previous experiments revealed the possibility of FEP substrate with a biopolymer porous pattern for cytocompatibility improvement. The application of the PLLA layer had a positive effect on the surface properties of the substrate, and the number of cells was significantly increased on the biopolymer microstructure compared to pristine FEP [54], in which we revealed that the PLLA pattern present on the treated FEP foil can be used for MRC-5 cell growth enhancement. The effect of the surface roughness on cell response depends strongly on the cell type and size [28]. Macroroughness (100 μm to 1 mm) can influence the behavior of larger cells (neurons) and usually does not restrict the adhesion and spreading of small cells [29]. On a micron and submicron scale (100 nm to 100 μm), many researchers have conflicting opinions. Some experiments show obvious cell adhesion and proliferation [30,31] and others describe the negative effect of the microroughness on the cell behavior [32,33]. Small cells, such as endothelial cells, are sensitive to surface nanoroughness (less than 100 nm), which demonstrably improves their adhesion and growth [34]. Therefore, a different type of polymer for hexagonal pattern formation was chosen for this study with a different type of cell, the interaction of which has been described. Different biomaterials have different requirements for cell adhesion despite the same surface roughness. There is no general trend that describes the relationship between the surface roughness and the cell response; it always depends on other physicochemical properties of the biomaterials and the specific cells [36]. The surface charge of the material strongly affects the cell behavior [37] and it can be regulated through the chemical functional groups in the polymer chains. The importance of surface chemistry was confirmed since the pristine substrate FEP did not show support of cell adhesion and proliferation for both osteoblasts (U-2 OS) and fibroblasts (MRC-5), which did not prefer unmodified polymer FEP due to its low surface wettability, absence of oxygen, and low surface roughness. The effect of plasma exposure was confirmed to be a significant tool for surface activation, also affecting the HCP formation significantly [6,47,48,49]. The subsequent process of a HCP formation from polystyrene led to the improvement of U-2 OS cell growth, which was previously confirmed for PLLA microstructure and human primary lung fibroblasts [54]. For future experiments, we see potential in the application of nanotextile perfluorinated substrates, which after plasma or laser activation should have excellent properties for following application of either biopolymer or “cell-friendly“ materials and which could serve as an excellent cell support.

## 5. Conclusions

We prepared a honeycomb-like pattern from polystyrene on plasma-treated perfluorinated polymer FEP. The plasma treatment was confirmed to play a crucial role in the pattern formation since the surface wettability and chemistry were altered significantly, and thus, the process of improved phase separation was successfully applied. The pattern aging was confirmed; more pronounced changes were observed for patterns constructed on a substrate that underwent plasma treatment with 8 W and 240 s, however, after a short time of aging, the process can be neglected with only minor contact angle changes. The surface morphology of the honeycomb pattern was confirmed using both atomic force microscopy and scanning electron microscopy. The pristine substrate FEP did not show support of cell adhesion and proliferation for both osteoblasts (U-2 OS) and fibroblasts (MRC-5), which did not prefer unmodified polymer FEP due to low surface wettability, absence of oxygen, and low surface roughness. We have confirmed that the plasma exposure itself of perfluorethylenepropylene significantly improved the cytocompatibility for both studied cell lines. The subsequent process of the honeycomb-like pattern formation from polystyrene led to the improvement of U-2 OS cell growth; the cells were able to mimic the pattern after the sixth day of growth. The success of U-2 OS growth and filopodia attachment was confirmed using scanning electron microscopy of the patterned area with grown cells. The osteoblasts were more successful in both adhesion and proliferation compared to the fibroblasts on the HCP. For MRC-5 cells, the plasma modification itself improved cell adaptation to the material and ability to grow to a high extent, however, from substrates with a HCP, the best results were confirmed for the sample pretreated using plasma at 3 W and 240 s. In addition, this experiment enables various material surfaces to be prepared with the possibility of drug release due to an extreme increase of an effective surface area for particular HCP-like structures.

## Data Availability

Data is contained within the article or supplementary material.
